# Task-Evoked Prefrontal Hemodynamics During Cognitive Assessment in Amyotrophic Lateral Sclerosis Using fNIRS

**DOI:** 10.3390/brainsci16080895

**Published:** 2026-08-21

**Authors:** Saqer Alshehri, Bartu Atabek, Terry Heiman-Patterson, Hasan Ayaz

**Affiliations:** 1 School of Biomedical Engineering, Science and Health Systems, Drexel University, Philadelphia, PA 19104, USA; 2 Department of Neurology, Lewis Katz School of Medicine, Temple University, Philadelphia, PA 19140, USA; 3 Department of Psychological and Brain Sciences, College of Arts and Sciences, Drexel University, Philadelphia, PA 19104, USA; 4 Drexel Solutions Institute, Drexel University, Philadelphia, PA 19104, USA; 5 A. J. Drexel Autism Institute, Drexel University, Philadelphia, PA 19104, USA; 6 Center for Injury Research and Prevention, Children’s Hospital of Philadelphia, Philadelphia, PA 19104, USA

**Keywords:** amyotrophic lateral sclerosis (ALS), functional near-infrared spectroscopy (fNIRS), neuroergonomics, cognitive assessment, prefrontal cortex (PFC), King-Devick Task (KDT)

## Abstract

**Highlights:**

**What are the main findings?**

- Despite comparable behavioral performance, wearable fNIRS revealed a group-specific hemodynamic pattern in ALS: deoxygenated hemoglobin (HbR) at the left DLPFC rose progressively with task difficulty in ALS participants but remained stable in controls, with significant between-group differences at the three harder conditions.
- Oxygenated hemoglobin (HbO) increased with task difficulty across all prefrontal optodes in both groups, confirming robust task-evoked engagement, so the ALS-specific effect was isolated to HbR regulation at the left DLPFC rather than a general activation difference.

**What are the implications of the main findings?**

- The dissociation between preserved behavioral performance and altered prefrontal hemodynamics suggests that routine behavioral measures may miss subtle cortical changes in ALS, which wearable fNIRS can reveal as an objective physiological readout during brief cognitive-motor assessment.
- Because fNIRS is portable, non-invasive, and tolerant of naturalistic settings, it may complement conventional clinical and behavioral tools for sensitive, repeatable monitoring of cognitive-motor function and, pending larger and longitudinal studies, potentially disease progression in ALS.

**Abstract:**

**Background/Objectives:** Amyotrophic lateral sclerosis (ALS) is a progressive neurodegenerative disease increasingly recognized for extra-motor manifestations, including cognitive dysfunction involving frontal cortical systems. Conventional clinical assessments are primarily behavioral and may not capture subtle alterations in the neural systems that support cognitive performance. This pilot study examined whether wearable functional near-infrared spectroscopy (fNIRS) could detect task-evoked prefrontal hemodynamic differences during the King-Devick Task (KDT), a rapid oculomotor number-naming task that engages visual scanning, attention, processing speed, and verbal response production. **Methods:** Sixteen participants, including seven individuals with ALS and nine age-matched healthy controls (HC), completed four progressively difficult KDT conditions while prefrontal cortical activity was recorded. **Results:** As task difficulty increased, response times became slower across participants, while accuracy remained preserved and behavioral performance did not differ significantly between groups. Prefrontal oxygenated hemoglobin (HbO) responses increased with task difficulty across all prefrontal optodes, supporting task-evoked cortical engagement. In contrast, deoxygenated hemoglobin (HbR) responses showed a group-specific pattern in the left dorsolateral prefrontal cortex: individuals with ALS demonstrated progressively increasing HbR responses across difficulty conditions, whereas HC showed relatively stable responses, with group differences emerging at higher difficulty levels. **Conclusions:** These findings suggest a dissociation between preserved behavioral performance and altered prefrontal hemodynamic regulation in ALS. The observed HbR pattern may reflect differences in cortical recruitment, neurovascular coupling, oxygen extraction, vascular responsiveness, or the efficiency of cortical resource allocation during increasing cognitive-motor demands. Wearable fNIRS may therefore complement behavioral assessments by revealing task-evoked neural alterations that are not evident from performance measures alone.

## 1. Introduction

Amyotrophic lateral sclerosis (ALS) is a progressive neurodegenerative disease characterized primarily by degeneration of upper and lower motor neurons, leading to progressive loss of voluntary motor control. Although ALS has historically been viewed as a motor neuron disease, it is increasingly recognized as a multisystem disorder with extra-motor manifestations, including cognitive and behavioral changes involving frontal cortical systems [1,2,3,4,5,6]. Cognitive involvement in ALS commonly affects executive function, attention, processing speed, language, and behavioral regulation, highlighting the need for assessment approaches that can capture changes beyond motor function alone.

Clinical and cognitive evaluations in ALS commonly rely on standardized behavioral and functional assessments. Tools such as the Edinburgh Cognitive and Behavioral ALS Screen (ECAS) and the ALS Cognitive Behavioral Screen (ALS-CBS) were developed to assess cognitive and behavioral symptoms while accounting for motor limitations in this population [7]. Similarly, the revised ALS Functional Rating Scale (ALSFRS-R) is widely used to assess functional status and disease progression [8]. These measures provide valuable clinical information, but they primarily characterize observable performance and functional ability. They do not directly measure the neural systems supporting cognitive performance and may therefore miss subtle alterations in cortical regulation when behavioral performance remains preserved. Neuroimaging approaches capable of measuring cortical function during cognitive assessment may therefore provide information that complements these behavioral measures.

Functional near-infrared spectroscopy (fNIRS) provides a non-invasive and wearable approach for monitoring task-evoked cortical hemodynamic responses [9,10,11]. By measuring changes in oxygenated hemoglobin (HbO) and deoxygenated hemoglobin (HbR), fNIRS can provide insight into prefrontal cortical engagement during cognitive task performance [12]. Its portability, tolerance of naturalistic testing environments, and compatibility with clinical and behavioral assessment paradigms make fNIRS particularly well suited for neuroergonomic studies of cognitive function in real-world or ecologically practical contexts [13,14,15]. Prior work has demonstrated the feasibility of fNIRS for measuring cognitive workload and task-related prefrontal activity in healthy adults and diverse clinical populations, including individuals with ALS [3,5]. Realizing this potential, however, depends on pairing fNIRS with a cognitive task that reliably engages the neural processes of interest while remaining brief enough for practical use in clinical settings.

The King-Devick Task (KDT) is a rapid oculomotor number-naming task that engages visual scanning, attention, processing speed, and verbal response production [16]. Because performance requires coordinated visual, cognitive, and motor processes, the KDT may be useful for probing cognitive-motor function in ALS. However, behavioral performance alone may not fully capture the neural demands required to maintain task performance, especially in individuals with neurological disease. Combining KDT performance measures with wearable fNIRS may therefore reveal task-evoked prefrontal changes that are not evident from response time (RT) or accuracy alone. Earlier ALS-fNIRS work has established the feasibility of measuring prefrontal activity in ALS during cognitive assessment, including work using the KDT [3,4]. The present study uses an entirely new and independent cohort and dataset, with no participant or data overlap with these earlier studies, and extends this prior work in four respects. First, that work derived a single total hemoglobin output per optode, summing HbO and HbR, whereas the present study analyzes the two chromophores separately, without which the pattern reported here is not recoverable. Second, the four KDT cards are treated as a parametric manipulation of cognitive-motor load and modeled as a Group × Condition interaction, characterizing how the prefrontal response scales with increasing demand rather than whether overall activation differs between groups. Third, the analysis is conducted at the level of individual optodes with false discovery rate correction across all sixteen prefrontal locations, so that any regional specificity is established under conservative control of multiple comparisons. Fourth, the neural comparison is evaluated under conditions in which behavioral performance was comparable between groups, using a brief and portable protocol suited to clinical settings.

Building on this earlier work, the present study investigated task-evoked prefrontal hemodynamic responses during the KDT in individuals with ALS and age-matched healthy controls (HC) using wearable fNIRS. We examined behavioral performance across progressively increasing task difficulty conditions together with changes in HbO and HbR over the prefrontal cortex (PFC). We hypothesized that individuals with ALS would show altered prefrontal hemodynamic responses during increasing task demands, potentially revealing neural differences even when behavioral performance is preserved. This multimodal approach may help characterize subtle changes in frontal cortical regulation in ALS and support the development of sensitive, minimally intrusive tools for cognitive monitoring.

## 2. Methods

### 2.1. Participants

Sixteen participants were recruited, including seven individuals with ALS (mean age = 61.43 years, SD = 7.59; five male and two female) and nine age-matched HC (mean age = 59.00 years, SD = 8.02; two male and seven female). All participants provided written informed consent under a protocol (#32421) approved by the Institutional Review Board of Temple University Lewis Katz School of Medicine and conducted in accordance with the Declaration of Helsinki. Exclusion criteria included an ALS-CBS score below 17, inability to see visual cues, a history of seizures or epilepsy, and inability to understand or speak English. All participants met the inclusion criteria and completed the data collection session. ALSFRS-R and ALS-CBS scores were used to characterize motor and cognitive performance in the ALS group (Table 1).

### 2.2. Experimental Protocol

**Figure 1:**
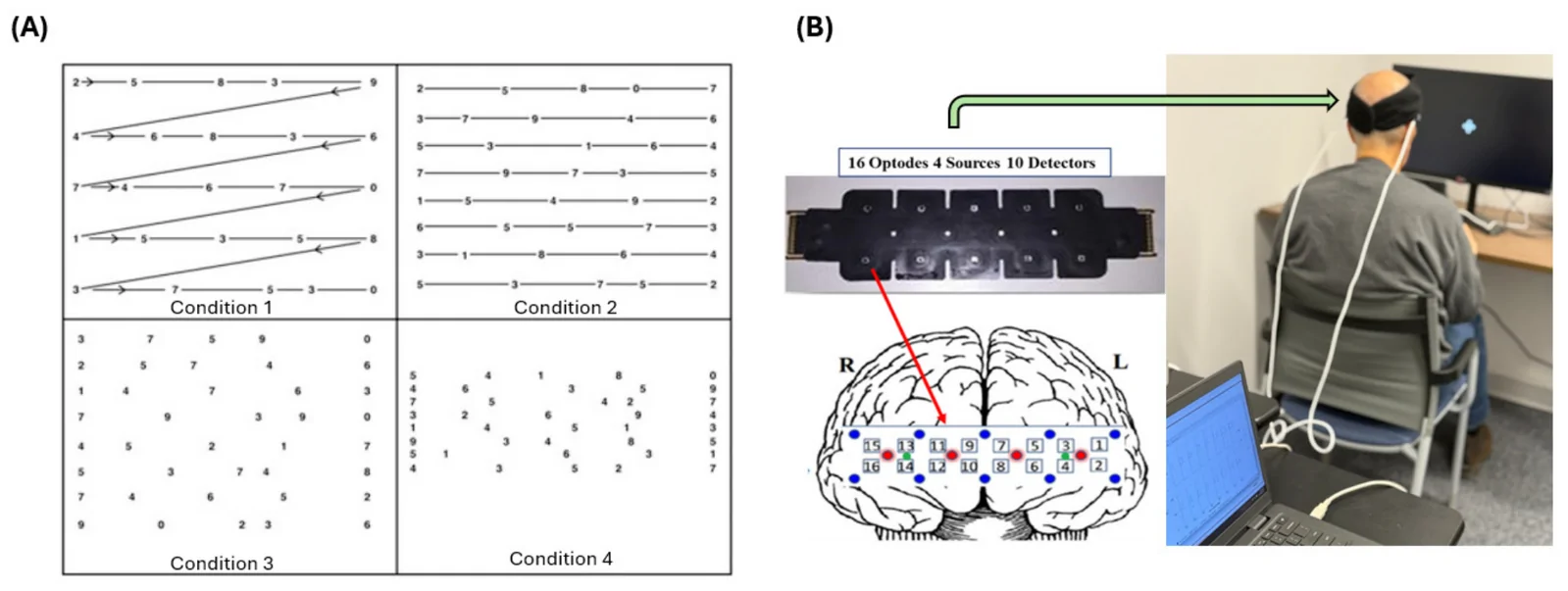
Experimental task and fNIRS setup. (**A**) Conditions for the King-Devick Task (KDT), with progressively increasing visual complexity from Condition 1 to Condition 4. (**B**) Prefrontal fNIRS data collection setup using a wearable and ultra-thin sensor pad with 16 measurement regions over the prefrontal cortex (PFC).

The KDT is a rapid oculomotor number-naming task that engages visual scanning, attention, processing speed, and verbal response production [17]. In this study, the task was administered on a computer monitor and consisted of one practice card and four test cards (task conditions), and the difficulty and visual complexity progressively increased from condition 1 to condition 4 similar to stimuli used in prior studies [17] (see Figure 1A). Participants were instructed to read the numbers on each card aloud, line by line, from left to right as quickly as possible [18]. An automated voice recording system was implemented within the task to capture participants’ responses for review and error verification. The average RT per condition was approximately 15 to 30 s [3], with a total task duration of approximately 3 min and no breaks between conditions. KDT was implemented using PsychoPy software (version 2023.2.2; www.psychopy.org) [19].

### 2.3. Data Acquisition and Preprocessing

During task administration, participants were seated upright and positioned approximately 40 to 50 cm from the display [20] to facilitate comfortable visual reading of the numbers presented on each condition. Behavioral outcomes derived from the KDT included RT for the full task, RT per condition in seconds, and accuracy. RT was defined as the duration from condition onset to the participant pressing a button after completing each condition.

Cerebral hemodynamics within the PFC were assessed using a continuous-wave fNIRS system. The fNIR Imager Model 2000S (fNIR Devices, LLC, Potomac, MD, USA) was used for all data acquisitions. Data acquisition was managed using COBI Studio Modern software (v1.2) [21]. The data collection setup included placement of a flexible, ultra-thin flat sensor pad on the participant’s forehead to provide bilateral PFC coverage (see Figure 1B). The system included a prefrontal optode array with four sources and ten detectors to capture sixteen measurement locations with a fixed source-detector separation of 2.5 cm and dual operational wavelengths of 730 nm and 850 nm, and an ambient light channel for noise correction [21]. Sensor placement followed a standardized protocol, with the lower optode row aligned along the supraorbital ridge and the array centered on the midline, providing consistent prefrontal coverage across participants. Optode locations for this array have previously been registered to MRI templates using a probabilistic, template-based spatial registration approach [22], visualized on brain surface renderings [23], and mapped to anatomical gyri and Brodmann areas [24]. Individual anatomical co-registration, optode digitization, and participant-specific MRI were not performed in the present study, and anatomical designations therefore reflect this template-based registration and the standardized sensor layout rather than individualized anatomy.

PFC activity was measured with fNIRS, and data preprocessing followed best practices in optical neuroimaging [25,26]. Raw light intensity signals were visually examined to identify motion artifacts and channels with low signal-to-noise ratio, and excessively noisy channels were excluded from further analysis, as outlined below and described before [27,28,29]. Signal processing was performed using fnirSoft (v4.11) [27]. To attenuate high-frequency noise and physiological artifacts, a 100th-order Hamming-windowed finite impulse response low-pass filter with a cutoff frequency of 0.1 Hz was applied [28]. Motion artifacts were addressed using the Sliding-Window Motion Artifact Rejection method (SMAR), and channel-level signal quality was assessed using a coefficient-of-variation based statistical measure [29]. Applying these criteria, 7% of optode-by-condition observations were excluded across the full dataset, with comparable rates in the two groups and no excess in the ALS group (ALS 5.4%, HC 8.3%). The modified Beer-Lambert law [30] was then used to transform optical density data into concentration changes in HbO and HbR. Because continuous-wave fNIRS does not yield absolute chromophore concentrations, the modified Beer-Lambert law expresses HbO and HbR as changes relative to a designated reference interval. The first five seconds of Condition 1 were used as this common reference point and were applied identically across all four KDT conditions, all optodes, and all participants.

### 2.4. Statistical Analysis

Statistical analyses were conducted to evaluate group differences in behavioral performance and prefrontal hemodynamic responses during the KDT. Descriptive statistics (mean ± standard deviation) were computed for all primary outcome measures, including RT, and fNIRS-derived HbO and HbR concentrations. For each participant, optode, and condition, a single HbO and HbR value was computed as the mean concentration change across that condition window, expressed relative to the common reference interval defined above. Neural data were analyzed primarily at the optode level, with each optode examined separately. Linear mixed-effects models were used to evaluate behavioral and fNIRS outcomes. Group (ALS vs. HC), Condition (Difficulty 1, 2, 3, and 4), and the Group × Condition interaction were modeled as fixed effects, and Subject was included as a random intercept to account for repeated measures. Separate models were estimated for RT, accuracy, HbO, and HbR. To correct for multiple comparisons across optodes, the false discovery rate (FDR) correction was applied using the Benjamini-Hochberg procedure [31]. Partial eta squared (η_p_^2^) was used to quantify effect sizes for fixed effects in linear mixed models (LME). All statistical analyses were performed using NCSS (v24.0.2), with statistical significance set at *p* < 0.05 (FDR-corrected).

## 3. Results

### 3.1. Behavioral Performance

**Figure 2:**
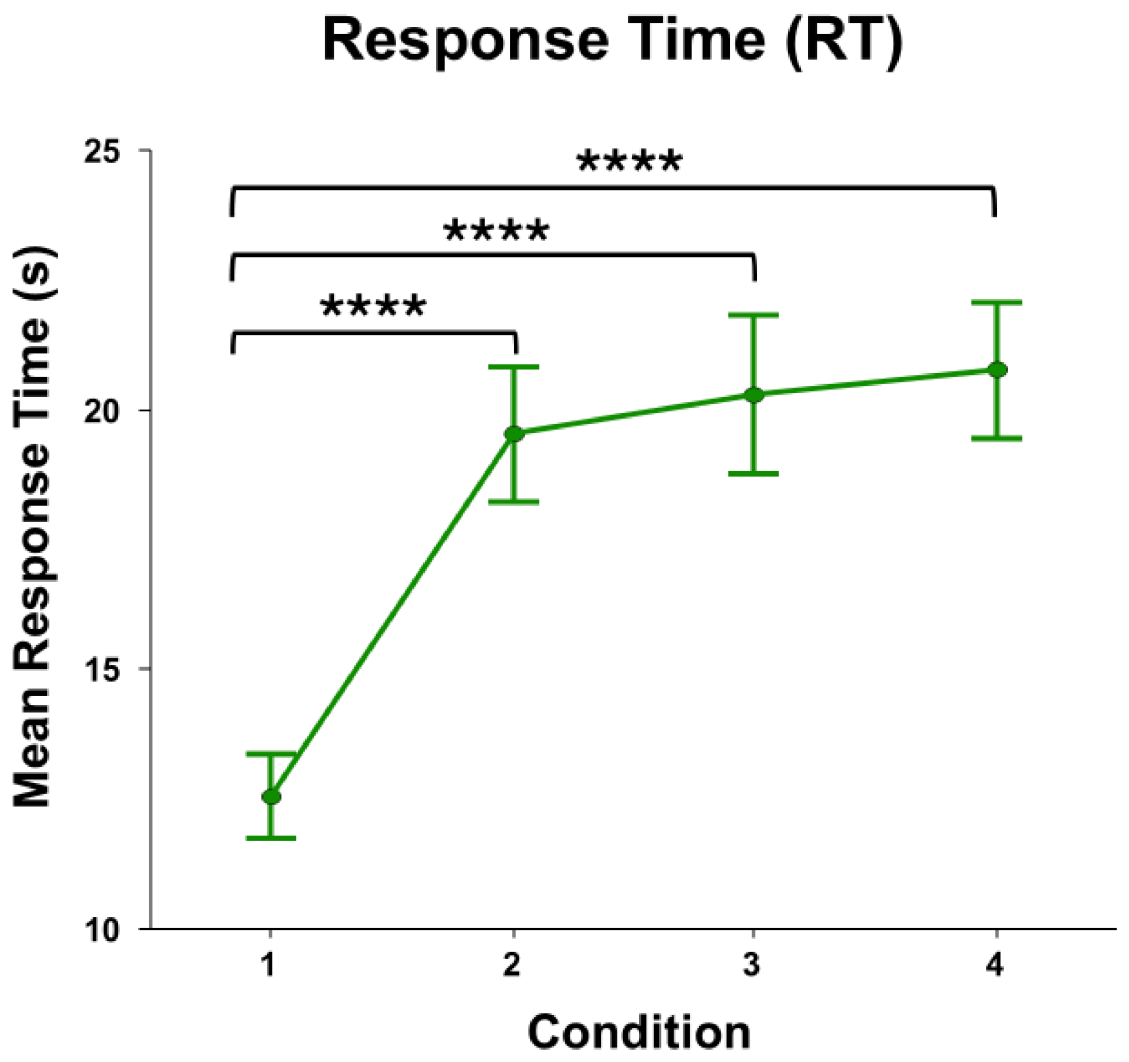
Mean response time (RT) across KDT conditions, collapsed across ALS and HC groups. RT increased significantly across conditions, indicating greater task demands as KDT difficulty progressed (main effect of Condition: F_3,42_ = 78.01, *p* < 0.0001, η_p_^2^ = 0.85). Brackets indicate Bonferroni-corrected post hoc comparisons relative to Condition 1; Conditions 2, 3, and 4 each differed significantly from Condition 1. Error bars represent standard error of the mean (SEM). **** *p* < 0.0001.

For behavioral measures, RT demonstrated a significant main effect of Condition (F_3,42_ = 78.01, *p* < 0.0001, η_p_^2^ = 0.85), indicating that RT increased as KDT difficulty progressed (Figure 2). Bonferroni-corrected post hoc comparisons relative to Condition 1 showed significantly longer RTs in Condition 2 (F_1,42_ = 128.34, *p* < 0.0001, η_p_^2^ = 0.75), Condition 3 (F_1,42_ = 161.40, *p* < 0.0001, η_p_^2^ = 0.79), and Condition 4 (F_1,42_ = 172.93, *p* < 0.0001, η_p_^2^ = 0.80).

No significant main effect of Group or Group × Condition interaction was observed. These findings indicate that task progression increased RT similarly in ALS and HC participants. Regarding accuracy, all participants achieved 100% accuracy across all conditions, with no errors reported in either group. Accuracy was therefore reported descriptively because there was no variance across participants or conditions.

### 3.2. fNIRS Findings

For HbO, a significant main effect of Condition was observed across all 16 optodes and survived FDR correction, indicating a consistent task-evoked hemodynamic response to increasing KDT difficulty throughout the PFC. No significant Group main effect or Group × Condition interaction survived correction, suggesting that the HbO response to task difficulty was comparable across the ALS and HC groups. This widespread HbO modulation supports participant engagement with the task and the feasibility of using the KDT with wearable fNIRS to assess cognitive-motor demand.

**Figure 3:**
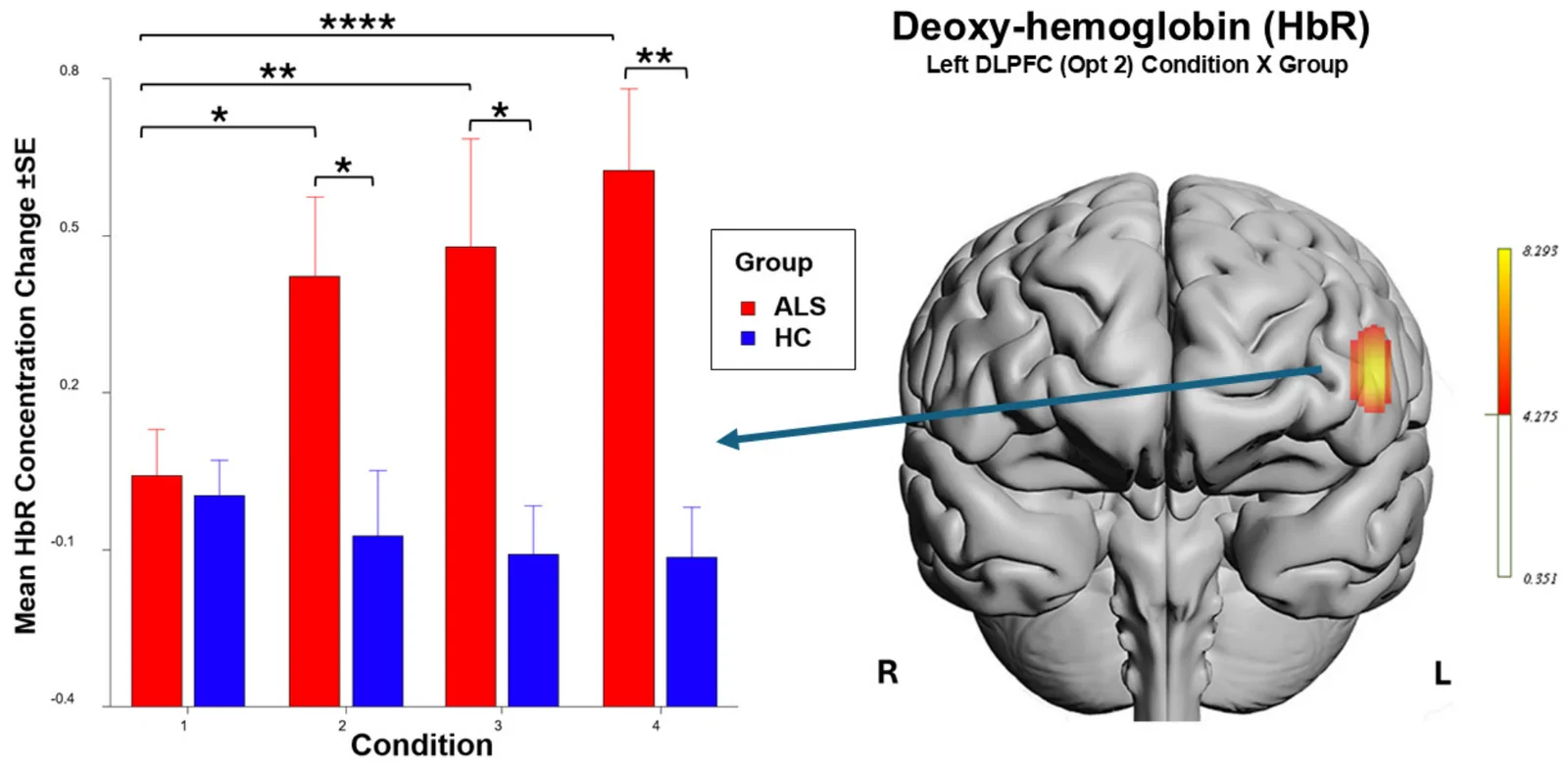
HbR concentration changes at the left DLPFC, Optode 2, by group across KDT conditions, with topographical representation of the significant area. Error bars represent SEM. Brackets indicate significant Bonferroni-corrected post hoc comparisons following the significant Group × Condition interaction. Within the ALS group, HbR increased significantly from Condition 1 to Conditions 2, 3, and 4. Between-group differences were significant at Conditions 2, 3, and 4. * *p* < 0.05, ** *p* < 0.005, **** *p* < 0.0001.

In contrast to the broadly comparable HbO response, HbR showed a group-specific pattern. A significant Group × Condition interaction was observed for HbR at Optode 2, corresponding to the left dorsolateral prefrontal cortex (DLPFC) region based on the sensor layout, and survived correction for multiple comparisons (F_3,41.1_ = 8.29, *p* < 0.001, η_p_^2^ = 0.38; FDR, q < 0.005). Main effects of Group and Condition at Optode 2 did not survive FDR correction, whereas the Group × Condition interaction did. This interaction indicated that while both groups showed task-evoked prefrontal responses, the pattern of HbR change across difficulty conditions diverged between groups. ALS participants showed a progressive rise in HbR at the left DLPFC as difficulty increased, whereas HC remained relatively stable, suggesting a different pattern of prefrontal hemodynamic regulation in ALS. Bonferroni-corrected post hoc comparisons were conducted to characterize the significant Group × Condition interaction at Optode 2. Within the ALS group, significant differences were revealed between Condition 1 and Condition 2 (F_1,41.1_ = 11.80, *p* < 0.05, η_p_^2^ = 0.22), Condition 1 and Condition 3 (F_1,41.1_ = 15.53, *p* < 0.005, η_p_^2^ = 0.27), and Condition 1 and Condition 4 (F_1,41.1_ = 27.71, *p* < 0.0001, η_p_^2^ = 0.40). While no significant differences were found within the HC group, significant between-group differences were observed at Condition 2 (F_1,24.7_ = 7.97, *p* < 0.05, η_p_^2^ = 0.24), Condition 3 (F_1,24.7_ = 11.21, *p* < 0.05, η_p_^2^ = 0.31), and Condition 4 (F_1,24.7_ = 17.73, *p* < 0.005, η_p_^2^ = 0.42) (see Figure 3).

Together, the fNIRS results showed two complementary patterns. HbO responses increased with KDT difficulty across the PFC, indicating robust task-evoked prefrontal engagement that was comparable between ALS and HC groups. In contrast, HbR showed a group-specific response at the left DLPFC, where individuals with ALS demonstrated progressively larger HbR changes as task difficulty increased, while HC participants showed relatively stable responses. Thus, despite preserved accuracy and comparable behavioral changes across conditions, ALS participants showed altered prefrontal HbR modulation under increasing cognitive-motor task demands. Complete mixed-effects model statistics for the Group, Condition, and Group × Condition effects on HbO and HbR responses at all sixteen optodes are provided in Supplementary Tables S1–S6.

## 4. Discussion

This study examined task-evoked prefrontal hemodynamic responses during the KDT in individuals with ALS and age-matched HC using wearable fNIRS. Behaviorally, increasing task difficulty was associated with slower RTs across participants, confirming that the task imposed progressively greater demands. However, individuals with ALS and HC showed comparable behavioral performance, with no significant group differences in response time or accuracy. In contrast, fNIRS revealed group-specific differences in prefrontal hemodynamic responses, particularly in HbR over the left DLPFC. Together, these findings suggest a dissociation between preserved behavioral performance and altered prefrontal hemodynamic regulation in ALS.

The behavioral findings indicate that both groups were able to complete the KDT successfully despite increasing task difficulty. RTs increased across the four difficulty conditions, consistent with the progressive visual and cognitive demands of the task. However, the absence of significant group differences in RT or accuracy suggests that individuals with ALS maintained performance at a level comparable to HC. This is important because routine clinical and behavioral measures may not fully capture subtle alterations in the neural systems supporting cognitive performance. In the context of ALS, where motor, speech, and cognitive symptoms may overlap, behavioral measures alone may provide an incomplete picture of task-related cognitive function.

The fNIRS findings provide complementary evidence that task performance was accompanied by robust prefrontal engagement. HbO showed a significant effect of task difficulty across prefrontal optodes, indicating that increasing KDT demands elicited measurable cortical hemodynamic responses. This broad HbO response supports the suitability of the KDT as a task capable of engaging prefrontal systems involved in attention, processing speed, and cognitive control. The presence of task-evoked HbO changes across the PFC also supports the feasibility of using wearable fNIRS to monitor cognitive task engagement in individuals with ALS.

The most notable finding was the group-specific HbR response over the left DLPFC. Individuals with ALS showed progressively increasing HbR responses across difficulty conditions, whereas HC showed relatively stable HbR responses. Importantly, this pattern emerged despite comparable behavioral performance between groups. Under typical task-evoked cortical activation, increased neural activity is often accompanied by increased cerebral blood flow and oxygen delivery, reflected by increased HbO and decreased HbR. Therefore, the increasing HbR response observed in the ALS group should not be interpreted simply as greater neural activation. Rather, it may reflect altered prefrontal hemodynamic regulation, potentially involving differences in neurovascular coupling, oxygen extraction, vascular responsiveness, or the efficiency of cortical resource allocation during increasing task demands.

One candidate interpretation is that individuals with ALS may engage different prefrontal regulatory mechanisms to maintain behavioral performance as task demands increase. In this view, preserved RT and accuracy may be achieved through greater physiological effort or altered cortical regulation that is not apparent from behavioral measures alone. The DLPFC is centrally involved in executive control, attentional regulation, working memory, and cognitive flexibility, all of which may contribute to successful performance during rapid visual scanning and number naming. The observed HbR pattern may therefore reflect increased vulnerability of frontal cortical systems in ALS during tasks that require sustained attention and efficient cognitive-motor coordination.

An alternative, and not mutually exclusive, interpretation is that the HbR pattern reflects altered neurovascular coupling in ALS. Cognitive dysfunction in ALS has been associated with frontal and frontotemporal involvement, and prior neuroimaging studies suggest that ALS can affect systems beyond motor pathways. If the coupling between neural activity, blood flow, and oxygen metabolism is altered, task-evoked hemodynamic responses may differ even when behavioral performance remains intact. From this perspective, the HbR increase in ALS may indicate a change in the balance between oxygen delivery and oxygen utilization during cognitive task performance. Future studies combining fNIRS with complementary measures, such as EEG, eye tracking, autonomic physiology, or clinical markers of disease progression, may help clarify whether these HbR changes reflect altered neural recruitment, vascular regulation, metabolic demand, or a combination of these mechanisms.

The KDT was administered in its standard, unmodified form, in which visual and cognitive complexity increase progressively from Condition 1 to Condition 4 without interposed rest. This fixed progression is intrinsic to the instrument as validated in concussion and traumatic brain injury populations [16,17,18], and counterbalancing the conditions or inserting rest intervals would depart from that validated administration. The cumulative demand arising from sustained visual scanning and continuous verbal output is therefore part of the load the paradigm is designed to impose rather than an incidental feature of it, and the question of interest is whether individuals with ALS sustain prefrontal hemodynamic regulation as that demand accumulates. Because condition order, task timing, and total recording duration were identical for both groups, generic influences such as fatigue, time on task, or slow signal drift would be expected to produce a main effect of Condition common to both groups. The group-specific divergence observed here cannot be generated by a confound shared equally across groups.

Because the KDT requires continuous verbal output, it is also important to consider whether speech, facial movement, respiration, or general motor effort could account for the observed group difference. Several features of the data argue against this. Response time on the KDT is the interval required to read each card aloud and is therefore a close proxy for speech production rate and sustained verbal motor output, yet it did not differ between groups, and all participants achieved 100% accuracy with responses verified against the automated voice recordings. Movement and systemic vascular influences also tend to be expressed broadly across channels and to produce correlated excursions in HbO and HbR, whereas the present effect was confined to a single optode after FDR correction across all sixteen locations and was restricted to HbR, with no corresponding group difference in HbO. Preprocessing further targeted these influences through motion artifact rejection and low-pass filtering. Taken together, these observations indicate that the observed pattern is unlikely to be attributable to peripheral speech, motor, or systemic contamination, although concurrent recording of systemic physiology would allow this to be demonstrated directly rather than inferred.

The dissociation between behavioral and neural measures is a central contribution of this study. Although standard behavioral outcomes suggested comparable task performance between groups, wearable fNIRS identified altered prefrontal hemodynamic responses in individuals with ALS. These findings suggest that wearable fNIRS may provide a complementary physiological readout during brief cognitive-motor assessments, potentially helping identify neural changes that are not captured by RT or accuracy alone. This finding highlights the potential value of neuroergonomic approaches that integrate objective neural measures with behavioral assessment. In clinical and translational settings, such measures may eventually prove useful for identifying subtle functional changes, although the present exploratory data do not support claims regarding clinical biomarkers, disease monitoring, or compensatory mechanisms. Because fNIRS is portable, non-invasive, and relatively tolerant of naturalistic testing environments, it may be particularly well suited for repeated monitoring in populations where conventional neuroimaging methods are less practical.

### Limitations and Future Work

Several limitations should be considered, and the study should be regarded throughout as exploratory. First, this was a pilot study with a small sample size, which limits statistical power and generalizability. ALS is a rare and rapidly progressive disease, and in-person neuroimaging research in this population is constrained by mobility, fatigue, and travel barriers, so the present sample is comparable in size to that of prior fNIRS studies in ALS. The findings should nonetheless be interpreted as preliminary and require replication in larger cohorts. Partial eta squared effect sizes are reported for all models so that the magnitude of the observed effects can be evaluated independently of statistical significance. Second, the ALS and HC groups were not balanced by sex composition. Because sex can influence cerebral hemodynamics and neurovascular coupling, this is a potential confound for the observed group difference in HbR, and we cannot exclude a contribution of sex to the divergent left DLPFC responses. Future studies should use larger, sex-balanced samples and, where adequately powered, model sex as a biological variable. Third, task conditions were administered in a fixed order of increasing difficulty as in the original KDT, without counterbalancing and without rest periods between conditions. As a result, parametric difficulty cannot be fully separated from time on task, stimulus order, or slow drift across the continuous recording. As noted above, these influences were identical for both groups and therefore cannot account for the divergence between them, but future work using counterbalanced or randomized condition orders with interposed rest periods would allow these contributions to be formally dissociated. Fourth, although the convergent evidence outlined above argues against a peripheral origin for the present effect, speech, bulbar, and oculomotor function were not measured directly in this study. Future work should include detailed measures of speech, bulbar function, eye movement control, and motor impairment to confirm the separation of cognitive from motor contributions to task performance. Fifth, fNIRS measurements were limited to the PFC and therefore cannot capture broader frontotemporal, motor, or subcortical network involvement. Sixth, HbR findings should be interpreted cautiously because fNIRS hemodynamic signals can reflect multiple physiological processes, including neurovascular coupling, oxygen extraction, systemic physiology, and vascular responsiveness. Systemic physiological signals such as heart rate, blood pressure, respiration, and skin conductance were not recorded, limiting the ability to separate cortical hemodynamic changes from systemic vascular contributions. Finally, longitudinal studies are needed to determine whether task-evoked prefrontal hemodynamic markers are sensitive to disease progression, cognitive decline, or treatment-related changes in ALS.

In summary, this study demonstrates the feasibility and potential value of wearable fNIRS for monitoring task-evoked prefrontal hemodynamic responses during cognitive assessment in ALS. Although individuals with ALS maintained behavioral performance comparable to HC, they showed altered HbR responses over the left DLPFC as task difficulty increased. These findings suggest that prefrontal hemodynamic measures may reveal changes in cortical regulation that are not evident from behavioral performance alone. Wearable fNIRS may therefore complement conventional clinical and behavioral assessments, although larger studies are required to establish whether such measures have clinical utility.

## 5. Conclusions

This pilot study investigated task-evoked prefrontal hemodynamic responses during the KDT in individuals with ALS using wearable fNIRS. Individuals with ALS maintained behavioral performance comparable to HC despite increasing task difficulty. However, fNIRS revealed altered HbR responses over the left DLPFC, suggesting that preserved task performance may be accompanied by altered prefrontal hemodynamic regulation.

These preliminary findings highlight a dissociation between behavioral performance and task-evoked cortical physiology in ALS. Wearable fNIRS may therefore complement conventional clinical and behavioral assessments by revealing neural alterations that are not evident from performance measures alone. Larger, sex-balanced, and longitudinal studies are needed to determine whether prefrontal hemodynamic markers can support sensitive monitoring of cognitive-motor function, disease progression, and treatment-related changes in ALS.

## Figures and Tables

**Table 1:** Demographic, clinical, and cognitive characteristics of participants with ALS.

#	Age	Gender	Onset Region	ALSFRS-R	ALS-CBS
1	58	F	R Arm	45	17
2	62	M	L Foot	26	19
3	67	M	Bulbar, R Arm	34	18
4	63	M	Legs	36	18
5	47	M	Bulbar	29	19
6	71	F	R Foot	40	17
7	62	M	Bulbar, L Leg	36	19
Mean	61.43	--	--	35.14	18.14
SD	7.59	--	--	6.39	0.90

## Data Availability

Data will be made available on request.

## Supplementary Materials

The following supporting information can be downloaded at: https://www.mdpi.com/article/10.3390/brainsci16080895/s1, Tables S1–S6: Mixed-effects model statistics for the main effects of Group and Condition and the Group × Condition interaction on HbO and HbR responses across all sixteen optodes.

## List of Abbreviations

ALS	Amyotrophic Lateral Sclerosis
ALS-CBS	ALS Cognitive Behavioral Screen
ALSFRS-R	Amyotrophic Lateral Sclerosis Functional Rating Scale-Revised
DLPFC	Dorsolateral Prefrontal Cortex
ECAS	Edinburgh Cognitive and Behavioral ALS Screen
FDR	False Discovery Rate
fNIRS	Functional Near-Infrared Spectroscopy
HbO	Oxygenated Hemoglobin
HbR	Deoxygenated Hemoglobin
HC	Healthy Controls
KDT	King-Devick Task
LME	Linear Mixed-Effects Model
MBLL	Modified Beer-Lambert Law
PFC	Prefrontal Cortex
RT	Response Time
SEM	Standard Error of the Mean
SMAR	Sliding-Window Motion Artifact Rejection
