# Supplementary material for: Task-Evoked Prefrontal Hemodynamics During Cognitive Assessment in Amyotrophic Lateral Sclerosis Using fNIRS
Source: Brain Sci. 2026 Aug 21;16(8):895. doi: 10.3390/brainsci16080895 (PMC13511391; doi:10.3390/brainsci16080895)
Supplement: Supplementary file 1 [file brainsci-16-00895-s001.zip › brainsci-4453073-supplementary.pdf]

SUPPLEMENTARY MATERIALS

for

Task-Evoked Prefrontal Hemodynamics During Cognitive Assessment in Amyotrophic Lateral Sclerosis Using fNIRS

Saqer Alshehri, Bartu Atabek, Terry Heiman-Patterson and Hasan Ayaz*

Table S1. Mixed-effects model statistics for the main effect of Group on HbO responses across optodes, with Subject as a random factor.

HbO	*Model Term*	*F-Value*	*Num DF*	*Denom DF*	*Prob Level*
Optode 1	Group	2.7272	1	13.9	0.120992
Optode 2	Group	1.0358	1	14.1	0.325969
Optode 3	Group	0.2042	1	14.1	0.658239
Optode 4	Group	1.0600	1	14.0	0.320665
Optode 5	Group	0.1670	1	13.1	0.689380
Optode 6	Group	6.1480	1	14.0	0.026445
Optode 7	Group	0.2893	1	12.0	0.600501
Optode 8	Group	5.7153	1	12.1	0.034007
Optode 9	Group	1.0464	1	12.1	0.326395
Optode 10	Group	0.4823	1	14.1	0.498701
Optode 11	Group	0.0007	1	14.1	0.979442
Optode 12	Group	0.4669	1	14.1	0.505540
Optode 13	Group	0.2650	1	11.1	0.616845
Optode 14	Group	1.4366	1	12.0	0.253734
Optode 15	Group	0.8515	1	12.1	0.374216
Optode 16	Group	2.4419	1	14.1	0.140345

Table S2. Mixed-effects model statistics for the main effect of Condition on HbO responses across optodes, with Subject as a random factor.

HbO	*Model Term*	*F-Value*	*Num DF*	*Denom DF*	*Prob Level*
Optode 1	Condition	6.7709	3	41.0	0.000817
Optode 2	Condition	4.9286	3	41.2	0.005133
Optode 3	Condition	5.9351	3	41.1	0.001844
Optode 4	Condition	16.1294	3	41.1	0.000000
Optode 5	Condition	7.3906	3	38.2	0.000507
Optode 6	Condition	19.6122	3	41.1	0.000000
Optode 7	Condition	7.9635	3	35.1	0.000352
Optode 8	Condition	13.8716	3	35.1	0.000004
Optode 9	Condition	4.7432	3	35.2	0.007008
Optode 10	Condition	7.2554	3	41.1	0.000514
Optode 11	Condition	7.5488	3	41.1	0.000391
Optode 12	Condition	5.7429	3	41.1	0.002235
Optode 13	Condition	8.4414	3	32.2	0.000280
Optode 14	Condition	5.4502	3	35.1	0.003490
Optode 15	Condition	4.4698	3	35.2	0.009243
Optode 16	Condition	8.1528	3	41.2	0.000224

Table S3. Mixed-effects model statistics for the Group × Condition interaction effect on HbO responses across optodes, with Subject as a random factor.

HbO	*Model Term*	*F-Value*	*Num DF*	*Denom DF*	*Prob Level*
Optode 1	GxC Int.	1.3702	3	41.0	0.265318
Optode 2	GxC Int.	0.9211	3	41.2	0.439142
Optode 3	GxC Int.	1.7884	3	41.1	0.164401
Optode 4	GxC Int.	4.0451	3	41.1	0.013108
Optode 5	GxC Int.	1.0185	3	38.2	0.395145
Optode 6	GxC Int.	3.5508	3	41.1	0.022444
Optode 7	GxC Int.	0.7062	3	35.1	0.554731
Optode 8	GxC Int.	2.7477	3	35.1	0.057370
Optode 9	GxC Int.	0.2569	3	35.2	0.855876
Optode 10	GxC Int.	0.4735	3	41.1	0.702397
Optode 11	GxC Int.	1.2030	3	41.1	0.320690
Optode 12	GxC Int.	0.8622	3	41.1	0.468351
Optode 13	GxC Int.	0.2337	3	32.2	0.872212
Optode 14	GxC Int.	1.6009	3	35.1	0.206634
Optode 15	GxC Int.	1.5332	3	35.2	0.222992
Optode 16	GxC Int.	1.9737	3	41.2	0.132903

GxC Int. = Group × Condition Interaction.

Table S4. Mixed-effects model statistics for the main effect of Group on HbR responses across optodes, with Subject as a random factor.

HbR	*Model Term*	*F-Value*	*Num DF*	*Denom DF*	*Prob Level*
Optode 1	Group	3.5350	1	13.5	0.081908
Optode 2	Group	9.4078	1	14.0	0.008334
Optode 3	Group	0.8018	1	14.1	0.385597
Optode 4	Group	6.4069	1	14.1	0.023924
Optode 5	Group	0.5321	1	13.2	0.478503
Optode 6	Group	2.5455	1	14.0	0.132847
Optode 7	Group	1.5637	1	12.1	0.234825
Optode 8	Group	0.0940	1	12.0	0.764402
Optode 9	Group	0.0166	1	12.0	0.899580
Optode 10	Group	0.1751	1	14.0	0.681975
Optode 11	Group	0.2875	1	14.1	0.600198
Optode 12	Group	0.1270	1	14.1	0.726803
Optode 13	Group	0.3588	1	11.1	0.561206
Optode 14	Group	2.5241	1	12.1	0.137996
Optode 15	Group	0.9392	1	12.0	0.351588
Optode 16	Group	1.8601	1	14.0	0.194064

Table S5. Mixed-effects model statistics for the main effect of Condition on HbR responses across optodes, with Subject as a random factor.

HbR	*Model Term*	*F-Value*	*Num DF*	*Denom DF*	*Prob Level*
Optode 1	Condition	5.4291	3	40.7	0.003101
Optode 2	Condition	3.1635	3	41.1	0.034478
Optode 3	Condition	0.9609	3	41.3	0.420261
Optode 4	Condition	2.8535	3	41.2	0.048792
Optode 5	Condition	0.6572	3	38.5	0.583385
Optode 6	Condition	2.2749	3	41.1	0.094129
Optode 7	Condition	0.7343	3	35.2	0.538583
Optode 8	Condition	3.6716	3	35.1	0.021183
Optode 9	Condition	0.7045	3	35.2	0.555726
Optode 10	Condition	7.1462	3	41.1	0.000569
Optode 11	Condition	0.4059	3	41.2	0.749511
Optode 12	Condition	4.6360	3	41.2	0.006975
Optode 13	Condition	0.9932	3	32.2	0.408379
Optode 14	Condition	3.8170	3	35.2	0.018153
Optode 15	Condition	1.8316	3	35.1	0.159352
Optode 16	Condition	2.1569	3	41.1	0.107739

Table S6. Mixed-effects model statistics for the Group × Condition interaction effect on HbR responses across optodes, with Subject as a random factor.

HbR	***Model Term***	***F-Value***	***Num DF***	***Denom DF***	***Prob Level***
Optode 1	GxC Int.	4.2503	3	40.7	0.010577
Optode 2	GxC Int.	8.2929	3	41.1	0.000198
Optode 3	GxC Int.	2.0704	3	41.3	0.118911
Optode 4	GxC Int.	2.9141	3	41.2	0.045574
Optode 5	GxC Int.	1.5160	3	38.5	0.225738
Optode 6	GxC Int.	0.8928	3	41.1	0.452954
Optode 7	GxC Int.	2.4060	3	35.2	0.083700
Optode 8	GxC Int.	0.4849	3	35.1	0.694929
Optode 9	GxC Int.	0.4824	3	35.2	0.696604
Optode 10	GxC Int.	0.3505	3	41.1	0.789004
Optode 11	GxC Int.	1.3476	3	41.2	0.272187
Optode 12	GxC Int.	0.7591	3	41.2	0.523510
Optode 13	GxC Int.	1.1015	3	32.2	0.362709
Optode 14	GxC Int.	2.0567	3	35.2	0.123683
Optode 15	GxC Int.	1.0218	3	35.1	0.394715
Optode 16	GxC Int.	0.9737	3	41.1	0.414392

GxC Int. = Group × Condition Interaction.
